# School Connectedness and Risk for Sexual Intercourse and Nonconsensual Sex in Adolescence

**DOI:** 10.1007/s11121-023-01635-w

**Published:** 2024-01-29

**Authors:** Chelsea R. Miller, Jamie M. Gajos, Karen L. Cropsey

**Affiliations:** 1 Department of Psychiatry and Behavioral Neurobiology, University of Alabama at Birmingham, 1720 7th Ave S, Office 909, Birmingham, AL 35233 USA; 2 Department of Family and Community Medicine, The University of Alabama at Birmingham, 1720 2nd Ave South, Birmingham, AL 35294-0110 USA; 3 Department of Psychiatry and Behavioral Neurobiology, University of Alabama at Birmingham, 1670 University Blvd., Volker Hall, Suite L107, Birmingham, AL 35233 USA

**Keywords:** Sexual victimization, School connectedness, Adolescence, Gender, Sexual activity

## Abstract

The CDC reports that 30% of high school students have engaged in sexual intercourse. Evidence suggests biological, personal, peer, societal, and family variables affect when a child will initiate sex. The school environment plays an important role in a child’s development. Evidence suggests that greater attachment to the school community can modify sexual risk-taking activity in adolescents. Future of Families and Child Wellbeing Study (FFCWS) comprises a cohort of approximately 4,700 families of children born in the U.S. between 1998–2000, over-sampled for non-marital births in large U.S. cities. Adolescents (*N* = 3,444 of 4,663 eligible) completed the wave six teen survey at approximately age 15. School connectedness was self-reported with four items measuring inclusiveness, closeness, happiness, and safety felt by the adolescent in their school environment. Sexual intercourse and nonconsensual sex were self-reported by the adolescent. Hierarchical regression analyses were conducted examining sexual intercourse, nonconsensual sex, risk factors, and school connectedness. In this sample of adolescents (48% female, 49% Black, 25% Hispanic, ages 14–19), school connectedness appears to reduce boys’ risk of nonconsensual sex (OR = 0.29, *p* < 0.01), and reduce girls’ risk of engaging in sexual intercourse (OR = 0.55, *p* < 0.01). Findings suggest gender differences in the association between school connectedness and sexual practices in adolescents. School connectedness may confer protection for boys’ risk of nonconsensual sex, and for girls’ risk of engaging in sexual intercourse. Further exploration of the relationship between school connectedness may allow for recommendations into preventative measures for teenage sexual behaviors.

## Introduction

Fostering connection and social relationships are innate human desires, but their absence can result in negative psycho-social outcomes (Baumeister & Leary, 1995). As children transition from childhood to adolescence, they begin to explore their place in the world as they form independent connections outside of the family structure (Lourenço, 2016). These connections have profound impacts on future social, health, and achievement outcomes (Raniti et al., 2022; Resnick et al., 1997; Rose et al., 2022; Weatherson et al., 2018). Children’s perceived sense of connectedness in school has the potential to influence many aspects of children’s lives, especially since children spend over 1000 h a year from age 5–18 in school settings.

Prior to the 1970’s, school was thought to have a marginal impact on children’s psychological development (Rutter & Maughan, 2002); however, in 1979, Bronfenbrenner published his model of ecological development which placed schools within a *microsystem* of interactions that impact human development (Lerner, 2002). He theorized that children are not solely genetically predestined to develop behaviors, but are also influenced by their larger environment, which he divides into a hierarchy of systems. At the center are the *individual traits* (gender, age, health) of the child, and how the child interacts at any given time with their *microsystem* (peers, family, teachers/school). The *exosystem* (school system, juvenile justice system, parents work, neighborhood) indirectly influences behavior through a *mesosystem* in which the *microsystem* and *exosystem* interact and directly impact development. All systems function under social and policy norms of the *macrosystem*, causing human development within the *chronosystem* of time (Lerner, 2002). In short, layers of interconnected environments affect human development.

Evidence suggests that the school environment impacts adolescent behavior both within and external to school (Chapman et al., 2013; Goodenow, 1993; Leonard, 2011). School connectedness, which is defined as feeling cared for, valued, accepted, and supported by fellow students and teachers (Goodenow, 1993) largely functions within Bronfenbrenner’s *microsystem* of direct interaction. Hence, the proximity of these interactions may have a significant impact on individual development. Literature already shows that perceived school connectedness promotes emotional wellbeing (Eugene, 2021; Langille et al., 2015; Marraccini & Brier, 2017; Raniti et al., 2022), improves academic achievement (Anderman, 2002), improves health outcomes (Steiner et al., 2019; Weatherson et al., 2018), and decreases delinquent behavior (Govender et al., 2013; Kearney, 2008; Weatherson et al., 2018); all of which will impact a child’s future developmental trajectory. However, standardized measures of school connectedness require further development (Hodges et al., 2018), though literature supports a multidimensional analysis of peer, teacher, and school interactions, to assess a child’s perceived sense of connectedness at school (Hodges et al., 2018; Maddox & Prinz, 2003).

At the center of Bronfenbrenner’s ecological framework of development is the *individual* with their innate characteristics, such as gender and age that affect how they interact with their *microsystem*. During middle childhood to adolescence, youth develop their sense of identity (Erikson, 1968) in which sexuality also emerges (Fortenberry, 2013). The CDC reports that 30% of high school students surveyed in 2021 engaged in sexual intercourse (CDC, 2021). An adolescent’s decision to engage in sex is a multifactorial interplay of biological, personal, peer, societal, and familial variables (Buhi & Goodson, 2007; Hofferth et al., 1987; Kar et al., 2015; Lee et al., 2018). Thus, it appears that not only individual characteristics may lead to an adolescent’s choice to engage in sexual activity, but also their closely related *microsystem* environments.

Current data shows that peers may have an influence on adolescent sexual behavior. A study showed that 33% of teens who had a sexually active friend, engaged in sex by the next year (Maxwell, 2002). This is consistent with studies showing that individuals may be more likely to engage in sex if their peers are also engaging in sex (Ali & Dwyer, 2011; Steele et al., 2020). Peer attitudes about permissive sexual behavior can increase risk of girls engaging in risky sexual behavior (multiple partners, early age of initiation, lack of contraception) (Steele et al., 2020), and teens may have double the risk of having sex if they perceive their peers to have favorable attitudes towards one night stands (Potard et al., 2008). Conversely, peer norms about abstaining from sex (Santelli et al., 2004) or delaying sex initiation (Carvajal et al., 1999) may be protective. Simply having friends correlated to girls having sex by grade 12, versus those who had less friends (Zimmer-Gembeck & Helfand, 2008). However, another study showed that boys with fewer friends were more likely to initiate sex early (Burke et al., 2018). But, boys may be more susceptible to social pressure than girls, based on a study where boys would respond more sexually explicitly to questions publicly (in an online chat forum) than what they report privately (Widman et al., 2016). Studies that rely on adolescents reporting their perceptions of their friend’s sexual behavior, may therefore, not accurately represent actual peer behavior.

Evidence supports that increased parental involvement may delay sexual debut (Buhi & Goodson, 2007; Longmore et al., 2009; Roche et al., 2005), but this impact may be small (Zimmer-Gembeck & Helfand, 2008). Two-parent households may delay sex initiation (Lammers et al., 2000; Zimmer-Gembeck & Helfand, 2008), and in studies evaluating family structure, a father’s presence seems to delay sex initiation (Jordahl & Lohman, 2009) and reduce risky sexual behavior (e.g., no condom use) (Guilamo-Ramos et al., 2012). Single mother households, even if the household is stable, can still lead to early sexual debut (Jordahl & Lohman, 2009; Steele et al., 2020). But, maternal education (Jordahl & Lohman, 2009) or family values on education (Gilliam et al., 2007) may delay sex initiation. The current study specifically looks at a sample of adolescents born to single-parent households, which may place them at increased risk of detrimental sexual behavior. However, some studies show that children with low family attachment, but higher school connectedness, are less likely to partake in risky behavior (Rovis et al., 2016; Wade & Brannigan, 1998). Therefore, it is within reason to consider that school—where children spend a significant portion of time and form key relationships—could also alter these sexual practices.

Previous research on the *microsystem* of school suggests that greater attachment to and success in school reduces sexual risk behaviors (Kirby, 2001) such as unprotected sex (Wilkins et al., 2023) and number of sexual partners (Rose et al., 2022). School connectedness could delay the onset of sexual activity, but individual choices (e.g., pledge of abstinence) may be more indicative of reduced sexual risk taking (Resnick et al., 1997; Santelli et al., 2004). Similar findings suggest that feeling cared for and connected to school delays sex initiation (Bonny et al., 2000; McNeely et al., 2010; Monahan et al., 2010). Early onset of sexual activity has also been linked to risker sexual behavior (Kuortti & Kosunen, 2009). For instance, students who engage in risky sexual behavior (e.g., multiple partners and lack of contraception) had lower GPAs, contemplated suicide more often, and had higher alcohol use, compared to those who have sex, but with lower risk (e.g., monogamy and contraception) (Luster & Small, 1994). Though the predominant stance in the literature is that adolescent sexual activity is a risk behavior, a growing body of literature supports a sex-positive view on teenage intercourse as a normal behavior that does not worsen psychological functioning (Harden, 2014). However, even these studies mention that among U.S. teenagers, there are exceptionally high rates of negative health outcomes (sexually transmitted disease and unintended pregnancy) compared to other countries (Harden, 2014).

Another negative health concern for U.S. teenagers is sexual violence, due to its prevalence and impact on the physical and mental health of victims. The CDC reports that 42.2% of first-reported sexual assault occurs in adolescence, of which the victims are predominantly female (Basile et al., 2014). In an independent survey of middle and high school students, 15% reported unwanted sexual experiences; of which boys reported peer pressure and regret as the cause, and girls reported forced sex and child abuse (Erickson & Rapkin, 1991). The National Youth Risk Behavior Survey showed female teenagers to be two-times as likely as male teenagers to be victims of forced sexual contact, and for females who were victims of forced sexual contact to engage in riskier sexual activity than their peers after the assault (Howard & Wang, 2005). Unwanted sexual contact is known to have a deleterious effect on mental health of victims across the lifespan, with an increase in future suicide attempts and self-injurious behaviors reported in girls with childhood sexual assault (Bentivegna & Patalay, 2022; Satyanarayana et al., 2015). Moreover, sexual violence is linked to increased reporting of post-traumatic stress symptoms, depression, anxiety, and eating disorders in adolescents (Banvard-Fox et al., 2020; Bentivegna & Patalay, 2022; Khadr et al., 2018; Satyanarayana et al., 2015).

The school environment plays an important role in children’s lives as they form interpersonal relationships and define the societal norms which they want to follow. Based on Bronfenbrenner’s model, schools are an environment of interconnected *microsystems* (peers, family, teachers) interacting and exerting developmental pressures on children. Focusing on the *microsystem* of school itself, we hypothesize that connectedness to the school community could reduce adolescent engagement in sexual activity, as well as be protective from non-consensual sex. Given the gender differences in unwanted sexual contact predominantly affects girls, this study will also explore gender differences in these variables among high-risk adolescents from the Future of Families and Child Wellbeing Study (FFCWS).

## Methods

### Data

Data were drawn from the FFCWS, comprising a cohort of approximately 4,700 families representative of a nationally-based sample of non-marital births in large U.S. cities (see Reichman et al. (2001) for additional study design details). Six waves of data are collected to date, beginning in 1998–2000, approximately 48 h after birth. Additional waves of data were collected via telephone-based interviews with mothers and fathers when the children were ages one (1999–2001), three (2001–2003), five (2003–2006), nine (2007–2010) and fifteen (2014–2017). De-identified data from the FFCWS are publicly available and approval for secondary data analyses was approved by the University of Alabama at Birmingham Institutional Review Board. The analytic sample for the current study is based on the sample of adolescents who completed the wave 6 survey through self-report at approximately age fifteen (Bendheim-Thoman Center for Research on Child Wellbeing, 2021). The analytic sample for the current study is based on the sample of adolescents (*N* = 3,444 of 4,663 eligible) who completed the wave 6 teen survey with complete data on study variables. The demographics of the adolescents included in the analytic sample include: 48% female, 49% Black, 25% Hispanic and ranged in age from 14–19 (*M*_*age*_ = 15.6).

## Measures

### Sexual Intercourse

Adolescents in a current relationship reported on their sexual behavior with the item, “Have you ever had sexual intercourse with [partner],—sometimes this is called ‘making love,’ ‘having sex,’ or “going all the way’?” (1 = *yes*, 0 = *no*). Next, adolescents who reported not currently being in a relationship and/or not having sex with their current partner were asked, “Have you ever had sexual intercourse with anyone, that is, made love, had sex, or gone all the way?” (1 = *yes*, 0 = *no*). Adolescents that responded ‘yes’ to either of these two items were coded as having had sexual intercourse.

### Nonconsensual Sex

Adolescents reported on their experiences with nonconsensual sex with the item, “Have you ever had sexual intercourse when you did not want to?” (1 = *yes*, 0 = *no*).

### School Connectedness

Four items adapted from the Panel Study of Income Dynamics Child Development Supplement (PSID-CDS-III, 2010) measure inclusiveness, closeness, happiness, and safety felt by the adolescent in their school environment. The items included, “I feel close to people at my school”, “I feel like I am part of my school”, “I am happy to be at my school”, and “I feel safe at my school.” The items were rated on a 4-point scale (3 = *Strongly Agree*, 0 = *Strongly Disagree)* and were averaged into an index representing greater school connectedness (α = 0.73).

### Adolescent Risk Factors

*Substance use.* Youth reported on the frequency of their past 30-day alcohol, cigarette, and cannabis use. Responses were dichotomized as: 0 = *no use*, 1 = *any past 30-day use*. *Low Self-Control.* Adolescents reported on six items from an abbreviated form of the Dickman’s impulsivity scale (Dickman, 1990) related to how they behaved or felt during the past four weeks with items such as: “Often, I don’t spend enough time thinking over a situation before I act” and “I often say and do things without considering the consequences.” Items were rated on a 4-point scale (3 = *Strongly Agree*, 0 = *Strongly Disagree*) and averaged to create an index of teen impulsivity (α = 0.78). *Parental Physical Abuse*. Adolescents self-reported on an item adapted from the Parent–Child Conflict Tactics Scale (CTSPC) (Straus et al., 1998). Teens reported on whether their mom/dad/primary caregiver “Hit or slapped you” in the past year, (1 = *yes*, 0 = *no*). *Delinquent Peers*. Adolescents self-reported on their friends’ engagement in delinquent activities during the past year. Eleven items developed by the FFCWS team assessed peer cigarette smoking, alcohol use, marijuana use, illegal or prescription drug use, invitations to drink together, giving or selling marijuana to the youth, damaging property, stealing something worth more than $50, using or threatening to use a weapon, selling marijuana or other drugs, and stealing something worth less than $50. All items were coded as 1 (i.e., *peer(s) engaged in the delinquent act in the past year*) or 0 (*peer(s) did not engage in the act during the past year*) and summed to reflect greater peer participation in delinquency (range 0–11).

### Covariates

*Extracurricular Activities.* Adolescents reported how often they spend time (e.g., never, less than once a month, at least once a month, once a week, or several times a week) on activities such as athletic or sports teams, clubs, religious services, etc. These items were averaged into a single score, where greater scores reflect more involvement in extracurricular activities (α = 0.59). *Teen employment.* Teens answered the question, “In the last 4 weeks, did you work – for pay – for anyone outside your home? This includes both regular jobs and things like baby-sitting or yard work”, (1 = *yes*, 0 = *no*). *Demographics.* Gender (1 = *male*, 0 = *female*), age (*M*_*age*_ = 15.6, *SD* = 0.77), and self-reported race/ethnicity (1 = *non-White*, 0 = *non-Hispanic White*), primary caregiver-reported annual household income (0 = *under $5,000* to 8 = *greater than $60,000*), primary caregivers’ highest level of education (0 = *less than high school* to 3 = *college or graduate*), and primary caregivers’ current marital status (0 = *not married*, 1 = *married*) were reported at year fifteen.

### Statistical Analyses

Logistic regressions were estimated in Stata 17 to predict Odds Ratios (ORs) to examine the associations between adolescent sexual intercourse and nonconsensual sex and study demographics. Next, a series of hierarchical logistic regression analyses were conducted to estimate the association between sexual intercourse/nonconsensual sex and teenage risk factors. Finally, the inclusion of school connectedness, extracurricular activities, and teen employment were estimated in the final model. All the hierarchical logistic regression models are reported separately by gender.

## Results

Descriptive statistics for study variables are reported in Table 1. Approximately 21% of the full sample reported engaging in sexual intercourse, with about 6% reported experiencing nonconsensual sex. Approximately 14% of girls reported engaging in sexual intercourse, whereas 28% of boys reported sexual intercourse and these differences between gender were significant (χ^2^(1) = 107.98, *p* < 0.001). Moreover, approximately 10% of girls and 5% of boys reported experiencing nonconsensual sex. Chi-square tests of independence also revealed significant differences by gender for nonconsensual sex (χ^2^(1) = 6.60, *p* = 0.010). Significant gender differences for past 30-day cigarette use (χ^2^(1) = 7.24, *p* = 0.007) and past 30-day cannabis use (χ^2^(1) = 9.91, *p* = 0.002) were also reported. Finally, significant mean differences for school connectedness (*t* = -5.11, *p* < 0.001) and engagement in extracurricular activities (*t* = 2.84, *p* = 0.002) were also reported by gender.

**Table 1:** Descriptive Statistics for Study Variables by Gender

	Full sample	Girls	Boys	
Variables	Mean (*SD*)	Mean (*SD*)	Mean (*SD*)	T-statistic/χ^2^
Age	15.60 (0.77)	15.59 (0.78)	15.61 (0.76)	-0.76
White	18.1%	18.3%	17.8%	0.13
Black	49.0%	49.7%	48.4%	0.59
Hispanic	24.9%	24.4%	25.4%	0.38
Other race	8.0%	7.5%	8.4%	0.89
Parent education	1.64 (0.98)	1.61 (0.98)	1.66 (0.98)	-1.40
Income	5.63 (2.54)	5.56 (2.56)	5.70 (2.51)	-1.59
Parent married (% yes)	41.3%	40.3%	42.2%	1.23
Sexual intercourse (% yes)	21.2%	13.7%	28.3%	107.98**
Nonconsensual sex (% yes)	6.3%	9.7%	4.7%	6.60**
Past 30-day alcohol use (% yes)	4.9%	4.4%	5.4%	1.62
Past 30-day cigarette use (% yes)	1.9%	1.3%	2.5%	7.24**
Past 30-day cannabis use (% yes)	8.0%	6.5%	9.4%	9.91**
Low self-control	1.47 (0.70)	1.45 (0.72)	1.49 (0.68)	-1.43
Physical abuse (% yes)	12.4%	11.9%	12.9%	0.77
Delinquent peers	1.70 (2.36)	1.68 (2.30)	1.72 (2.42)	-0.53
School connectedness	2.43 (0.58)	2.38 (0.60)	2.48 (0.56)	-5.11**
Extracurricular activities	1.25 (0.85)	1.29 (0.88)	1.21 (0.82)	2.84**
Teen employment (% yes)	28.3%	27.9%	28.8%	0.37

*SD* standard deviation

*p ≤ 0.05; **p ≤ 0.01

The hierarchical logistic regression models for predicting risk for sexual intercourse in girls and boys are presented in Tables 2 and 3, respectively. Table 2, Model 1 reports the association between risk for girls to engage in sexual intercourse and demographic covariates. Age was significantly associated with an increased risk of girls’ sexual intercourse (OR = 1.68, *p* < 0.001), whereas higher levels of parental education (OR = 0.81, *p* = 0.020) and households with married parents (OR = 0.55, *p* = 0.003) were associated with reduced risk of girls’ sexual intercourse. Next, teenage risk factors were added to Model 2. Results revealed previous 30-day cannabis use (OR = 10.26, *p* < 0.001), adolescent low self-control (OR = 2.18, *p* < 0.001), and delinquent peers (OR = 1.23, *p* < 0.001) were significantly associated with an increased risk of girls’ sexual intercourse. Moreover, these teenage risk factors remained significant in Model 3; however, greater school connectedness was associated with a reduced risk for girls’ sexual intercourse (OR = 0.55, *p* < 0.001).

**Table 2:** Association between Girls' Sexual Intercourse, Teen Risk Factors, and School Connectedness

	Model 1	Model 2	Model 3
	OR (95% CI)	OR (95% CI)	OR (95% CI)
Age	1.68** (1.39, 2.04)	1.82** (1.44, 2.30)	1.75** (1.38, 2.22)
Race/ethnicity	1.00 (0.63, 1.59)	1.13 (0.64, 1.98)	1.14 (0.64, 2.03)
Parent education	0.81* (0.68, 0.97)	0.86 (0.70, 1.07)	0.89 (0.71, 1.10)
Income	0.99 (0.92, 1.07)	1.00 (0.92, 1.09)	1.02 (0.93, 1.11)
Parent married	0.55** (0.38, 0.82)	0.69 (0.44, 1.07)	0.74 (0.47, 1.18)
Alcohol use	–	1.06 (0.49, 2.31)	1.06 (0.49, 2.33)
Cigarette use	–	4.85 (0.92, 25.55)	3.15 (0.57, 17.44)
Cannabis use	–	10.26** (5.53, 19.05)	9.16** (4.88, 17.18)
Low self-control	–	2.18** (1.60, 2.97)	1.98** (1.44, 2.71)
Parental abuse	–	1.62 (0.96, 2.72)	1.59 (0.94, 2.69)
Delinquent peers	–	1.23** (1.13, 1.33)	1.23** (1.13, 1.33)
School connectedness	–	–	0.55** (0.40, 0.75)
Extracurriculars	–	–	0.86 (0.68, 1.11)
Employment	–	–	1.17 (0.76, 1.80)
	*n* = 1,268	*n* = 1,260	*n* = 1,242

Significant findings are presented in bold

*OR* Odds ratio

*p ≤ 0 .05; **p ≤ 0.01

**Table 3:** Association between Boys' Sexual Intercourse, Teen Risk Factors, and School Connectedness

	Model 1	Model 2	Model 3
	OR (95% CI)	OR (95% CI)	OR (95% CI)
Age	2.04** (1.73, 2.41)	1.91** (1.59, 2.28)	1.83** (1.52, 2.20)
Race/ethnicity	1.95** (1.29, 2.98)	1.77** (1.13, 2.77)	1.97** (1.24, 3.13)
Parent education	0.87* (0.76, 1.00)	0.90 (0.77, 1.05)	0.91 (0.78, 1.06)
Income	0.92** (0.87, 0.97)	0.91** (0.86, 0.97)	0.92** (0.87, 0.98)
Parent married	0.60** (0.45, 0.80)	0.57** (0.42, 0.78)	0.57** (0.41, 0.78)
Alcohol use	–	1.59 (0.78, 3.23)	1.53 (0.75, 3.12)
Cigarette use	–	5.12** (1.42, 18.46)	5.09** (1.39, 18.58)
Cannabis use	–	3.20** (1.96, 5.23)	3.37** (2.04, 5.55)
Low self-control	–	1.11 (0.90, 1.37)	1.09 (0.87, 1.35)
Parental abuse	–	0.96 (0.64, 1.43)	0.93 (0.61, 1.39)
Delinquent peers	–	1.22** (1.14, 1.30)	1.20** (1.13, 1.28)
School connectedness	–	–	1.01 (0.78, 1.31)
Extracurriculars	–	–	0.92 (0.77, 1.00)
Employment	–	–	1.91** (1.41, 2.59)
	*n* = 1,369	*n* = 1,361	*n* = 1,338

Significant findings are presented in bold

*OR* Odds ratio

*p ≤ 0 .05; **p ≤ 0.01

Estimates for boys’ risk of sexual intercourse are presented in Table 3. Model 1 revealed that older (OR = 2.04, *p* < 0.001) and non-White boys (OR = 1.95, *p* = 0.002) were at an increased risk of engaging in sexual intercourse. However, higher parental education (OR = 0.87, *p* = 0.052), greater household income (OR = 0.92, *p* = 0.002), and having parents who were married (OR = 0.60, *p* = 0.001) reduced the odds of sexual intercourse in boys. Including teenage risk factors in Model 2 revealed that past 30-day cigarette use (OR = 5.12, *p* = 0.013), cannabis use (OR = 3.20, *p* < 0.001), and delinquent peers (OR = 1.22, *p* < 0.001) increased the risk for sexual intercourse in boys. These teenage risk factors remained significant in Model 3; additionally, teenage employment was associated with an increased risk for sexual intercourse in boys (OR = 1.91, *p* < 0.001).

The hierarchical logistic regression results for girls’ and boys’ risk for nonconsensual sex are presented in Tables 4 and 5, respectively. In Table 4, Model 1 shows that higher levels of parental education were significantly associated with an increased risk for girls’ nonconsensual sex (OR = 1.99, *p* = 0.048), whereas higher household income was associated with lower risk (OR = 0.77, *p* = 0.015). These demographic covariates remained significant in Models 2 and 3, although none of the teenage risk factors (nor school connectedness, extracurricular activities, or teenage employment) were associated with risk for nonconsensual sex in girls.

**Table 4:** Association between Girls' Nonconsensual Sex, Teen Risk Factors, and School Connectedness

	Model 1	Model 2	Model 3
	OR (95% CI)	OR (95% CI)	OR (95% CI)
Age	1.31 (0.70, 2.45)	1.55 (0.78, 3.09)	1.65 (0.81, 3.36)
Race/ethnicity	0.72 (0.17, 3.06)	0.52 (0.11, 2.51)	0.49 (0.09, 2.68)
Parent education	1.99* (1.01, 3.95)	2.08* (1.04, 4.17)	2.12* (1.03, 4.35)
Income	0.77* (0.62, 0.95)	0.76* (0.60, 0.96)	0.75* (0.59, 0.96)
Parent married	3.00 (0.95, 9.46)	2.82 (0.85, 9.44)	2.51 (0.73, 8.59)
Alcohol use	–	0.68 (0.10, 4.65)	0.60 (0.08, 4.49)
Cigarette use	–	4.43 (0.57, 34.50)	4.02 (0.46, 35.43)
Cannabis use	–	0.31 (0.06, 1.50)	0.29 (0.06, 1.41)
Low self-control	–	1.08 (0.39, 3.03)	1.05 (0.38, 2.96)
Parental abuse	–	1.90 (0.50, 7.27)	2.18 (0.54, 8.85)
Delinquent peers	–	1.04 (0.82, 1.33)	1.02 (0.79, 1.30)
School connectedness	–	–	0.93 (0.43, 2.02)
Extracurriculars	–	–	0.91 (0.46, 1.80)
Employment	–	–	2.16 (0.65, 7.18)
	*n* = 169	*n* = 167	*n* = 163

Significant findings are presented in bold

*OR* Odds ratio

*p ≤ 0 .05; **p ≤ 0.01

**Table 5:** Association between Boys' Nonconsensual Sex, Teen Risk Factors, and School Connectedness

	Model 1	Model 2	Model 3
	OR (95% CI)	OR (95% CI)	OR (95% CI)
Age	1.53 (0.88, 2.68)	1.55 (0.85, 2.82)	1.66 (0.90, 3.06)
Race/ethnicity	0.44 (0.11, 1.71)	0.37 (0.09, 1.52)	0.28 (0.06, 1.30)
Parent education	1.26 (0.69, 2.30)	1.51 (0.78, 2.93)	1.48 (0.76, 2.91)
Income	1.04 (0.83, 1.30)	1.02 (0.81, 1.28)	1.04 (0.84, 1.30)
Parent married	1.22 (0.40, 3.71)	1.36 (0.43, 4.23)	1.38 (0.41, 4.64)
Alcohol use	–	0.56 (0.10, 3.13)	0.63 (0.12, 3.46)
Cigarette use	–	3.31 (0.68, 16.26)	4.14 (0.82, 20.87)
Cannabis use	–	1.10 (0.30, 4.03)	1.60 (0.40, 6.31)
Low self-control	–	1.43 (0.60, 3.41)	1.28 (0.50, 3.25)
Parental abuse	–	0.65 (0.14, 3.10)	0.54 (0.10, 2.83)
Delinquent peers	–	1.07 (0.87, 1.32)	1.05 (0.85, 1.29)
School connectedness	–	–	0.29** (0.13, 0.66)
Extracurriculars	–	–	2.16* (1.03, 4.53)
Employment	–	–	1.07 (0.32, 3.63)
	*n* = 370	*n* = 366	*n* = 357

Significant findings are presented in bold

*OR* Odds ratio

*p ≤ 0 .05; **p ≤ 0.01

The results for boys’ risk for nonconsensual sex are presented in Table 5. None of the demographic covariates in Model 1, nor any of the teenage risk factors presented in Model 2, were significantly associated with risk for boys’ nonconsensual sex. However, Model 3 revealed that greater levels of school connectedness were associated with reduced risk for boys’ nonconsensual sex (OR = 0.29, *p* = 0.003), whereas extracurricular activities were associated with an increased risk for boys to report experiencing nonconsensual sex (OR = 2.16, *p* = 0.041).

## Discussion

School connectedness may reduce the risk of early initiation of sex in adolescents (Bonny et al., 2000; Foster et al., 2017; McNeely et al., 2010). Our findings suggest that school connectedness is associated with reduced risk for sexual intercourse among girls, but not for boys. However, school connectedness was associated with reduced risk for nonconsensual sex among boys, but not girls. Although boys reported higher levels of school connectedness than girls in this sample, other factors—such as family and peers—may also play a prominent role in overall sexual practices for male adolescents. This is supported by Bronfenbrenner’s model of ecological development in which *microsystems* act directly on child development, in which sexual practices emerge in adolescence. Familial connectedness, for instance, is central to emotional wellbeing in children (Eugene, 2021; Glover et al., 1998) and evidence shows family connectedness to be a predictor of later sex initiation (Hofferth et al., 1987; Markham et al., 2003; Miller, 2002; Resnick et al., 1997; Roche et al., 2005). For boys, higher family income and having married parents was associated with less risk of reporting sexual intercourse across all models, consistent with prior studies (Gilliam et al., 2007; Lammers et al., 2000; Steele et al., 2020). For girls, none of the familial demographics were associated with risk for sexual intercourse once peer and school variables were included in the model, suggesting that other social bonds outside of the home, and potentially within their *microsystem*, may be more influential for girls’ sexual activity.

Early adolescence is the age of highest influenceability and conformity to peer groups (Goodenow, 1993). Delinquent peers were associated with greater risk of engaging in sex for both girls and boys. This is consistent with findings that peer influence can be significant in adolescent engagement in consensual sex (Ali & Dwyer, 2011; Maxwell, 2002; Potard et al., 2008), while other studies report that peer behavior is not significantly influential (Hofferth et al., 1987; Resnick et al., 1997; Santelli et al., 2004). Further, boys reporting use of cigarettes and cannabis had a greater likelihood of engaging in sexual intercourse, whereas cannabis use was the only substance use risk for girls’ sexual intercourse. This is consistent with reporting that drug use is a risk factor for engagement in sexual activity (Burke et al., 2018; Cho & Yang, 2023; Santelli et al., 2004). For girls, low self-control was associated with a greater risk of reporting sexual intercourse. However, school connectedness did not significantly reduce these risk factors for girls. This may show how *individual* traits may outweigh environmental pressures of development on the decision to have sex. Employment was also associated with greater risk for boys’ sexual intercourse. Indeed, employment could expose children to older adolescents or adults, who may be more likely to engage in sex. This is consistent with evidence of middle schoolers who report having an older significant other to be more likely to report sexual activity (Marín et al., 2000). Furthermore, prior work found adolescents working greater than 20 h a week increased substance use (Resnick et al., 1997), of which substance use was associated with an increased risk of sexual intercourse for both genders in this study.

We also examined the link between school connectedness and nonconsensual sex. For girls, higher levels of parental education and lower family income were associated with increased risk for nonconsensual sex. Lower family income has been linked to increased sexual risk taking, such that teens will engage in sex to attain financial comfort (Anyanwu et al., 2020), or engage in sex if they have less supervised time inside of their home (Ethier et al., 2016; Longmore et al., 2009; Roche et al., 2005), or come from a single-parent household where they observe normalized sexual practices in their parent (Hofferth et al., 1987; Rossi, 1997; Steele et al., 2020). Perhaps girls from lower income families have less supervision as parents try to obtain work, placing them at risk of victimization (e.g., nonconsensual sex). Increased parental education seems contradictory to the finding that lower income increases nonconsensual sex in girls, as education is usually positively correlated to income, and education has been shown to reduce consensual sex in teens (Ethier et al., 2016; Hofferth et al., 1987; Jordahl & Lohman, 2009). However, girls from homes with greater education levels could be more likely to report nonconsensual sex if they receive education on the role of consent from their parents. Or children’s families with high education may have parents trying to attain career success, placing the children in the care of others outside of the home where they are also more likely to be victimized. This would be the same reason as low-income, simply put, time away from the nuclear family may confer risk of victimization.

When examining the risk for boys reporting nonconsensual sex, school connectedness was associated with decreased risk of nonconsensual sex and extracurricular activities were associated with an increased risk of reporting nonconsensual sex. The finding related to extracurricular activities may seem contradictory, as increased engagement in extracurriculars usually promotes school connectedness (Bonny et al., 2000). However, like employment for boys, extracurriculars may expose students to older peers. Although our measure of extracurricular activities does not *only* include sports in this study, there is a growing body of evidence linking sports participation to sexual victimization (Cheever & Eisenberg, 2022). Studies have found that sports-involved youth are more likely to be a victim of sexual violence, and males highly involved in sports are more likely to be the victim of school-based sexual harassment and coercion into sex than their non-sport peers (Cheever & Eisenberg, 2022). However, there is some evidence that having caring adults in the school can protect against sexual harassment (Doty et al., 2017), and the current study shows that school connectedness may confer some protection for boys against nonconsensual sex.

This study is not without limitations. First, our measure of connectedness is limited by the FFCWS that asked four questions generalized to the school community. Though the exact measurement of school connectedness is yet to be agreed on, the 35-item Student Engagement Instrument (SEI) is thought to have the strongest psychometric properties (Hodges et al., 2018). The SEI focuses on three components of teacher support relationships, peer support measures, and cognitive enhancement measures, of which we are missing in our limited survey of connectedness. Though peer support is an operational component of school belonging (Rutter & Maughan, 2002) there is strong evidence suggesting the quality of teacher-student relationships increases connectedness (Bonny et al., 2000; Chapman et al., 2013; Goodenow, 1993; Hawkins et al., 1999; McNeely et al., 2002). Moreover, students who perceive their teachers as having high expectations of them—a component of school connectedness—are less likely to engage in delinquent behavior (McNeely et al., 2002), which is also a risk for sexual activity. Future research focusing on teacher specific qualities would be beneficial to explore. Second our measure of nonconsensual sex is also a limitation, as there were no follow-up questions administered to understand the context of the reporting. Also, it is assumed that the individual reporting is a victim of non-consensual sex and not the perpetrator. Moreover, the data is self-reported, which comes with limitations as over or under reporting can occur. The content of this study is sensitive in nature, and thus, the adolescents may have withheld their reporting of sexual activity, victimization, and drug use. Furthermore, adolescents reported on peer behavior, which may not be an accurate representation of the actual activity of their peers. Finally, the variables in this study are associations and not causations. Adolescent sexual practices are complex, and there are likely other factors that are not currently considered in our models.

## Conclusions

The findings of this study support gender differences in the association between school connectedness and sexual practices in adolescents. Based on the findings of this study, *individual* attributes and interactions of *microsystems* may play a role in adolescents’ engagement in consensual sex, and *microsystems* may have a role in a child’s chance of nonconsensual sex. These results have implications for entities within the *microsystem* such as practitioners and educators. What might be viewed as protective behaviors in psychosocial development—such as engaging in extracurricular activities—might make boys more susceptible to engaging in sex and being victims of nonconsensual sex when participating in activities that remove them from the home. Though sexual victimization is reported in girls at a higher rate than boys, the evidence from this study supports unbiased screening for sexual victimization in adolescents regardless of gender. This study would also support screening for victimization during sports physicals, especially during those that are offered for free, at school, in lower income communities with health disparities (Burton et al., 2022).

School connectedness seems to confer some protection for boys’ risk of nonconsensual sex, and for girls’ risk of engaging in sexual intercourse. Sexual practices and victimization both have lasting outcomes on the physical and mental health of youth. Therefore, the school environment may impact adolescents’ lives beyond their education, and schools should continue to promote environments where students feel valued and accepted. Finally, since evidence has shown that the student–teacher relationships might have the biggest influence on psychosocial outcomes in students, further exploration of this relationship may allow for better recommendations into preventative measures for teenage sexual behaviors.

## Data Availability

Currently, Baseline, Year 1, Year 3, Year 5, Year 9, and Year 15 survey data are available to the public through the Office of Population Research data archive.
